# Integrating graph convolutional networks with large language models for structured biomedical material knowledge representation

**DOI:** 10.1093/rb/rbaf083

**Published:** 2025-08-09

**Authors:** Mufei Li, Yan Zhuang, Yao Hou, Ke Chen, Lin Han, Kefeng Wang, Xiangfeng Li, Xiangdong Zhu, Mingli Yang, Guangfu Yin, Jiangli Lin, Xingdong Zhang

**Affiliations:** College of Biomedical Engineering/National Engineering Research Centre for Biomaterials, Sichuan University, Chengdu 610065, China; College of Biomedical Engineering/National Engineering Research Centre for Biomaterials, Sichuan University, Chengdu 610065, China; College of Biomedical Engineering/National Engineering Research Centre for Biomaterials, Sichuan University, Chengdu 610065, China; College of Biomedical Engineering/National Engineering Research Centre for Biomaterials, Sichuan University, Chengdu 610065, China; Provincial Engineering Research Center for Biomaterials Genome of Sichuan, Sichuan University, Chengdu 610065, China; College of Biomedical Engineering/National Engineering Research Centre for Biomaterials, Sichuan University, Chengdu 610065, China; College of Biomedical Engineering/National Engineering Research Centre for Biomaterials, Sichuan University, Chengdu 610065, China; Provincial Engineering Research Center for Biomaterials Genome of Sichuan, Sichuan University, Chengdu 610065, China; College of Biomedical Engineering/National Engineering Research Centre for Biomaterials, Sichuan University, Chengdu 610065, China; Provincial Engineering Research Center for Biomaterials Genome of Sichuan, Sichuan University, Chengdu 610065, China; College of Biomedical Engineering/National Engineering Research Centre for Biomaterials, Sichuan University, Chengdu 610065, China; Provincial Engineering Research Center for Biomaterials Genome of Sichuan, Sichuan University, Chengdu 610065, China; College of Biomedical Engineering/National Engineering Research Centre for Biomaterials, Sichuan University, Chengdu 610065, China; Provincial Engineering Research Center for Biomaterials Genome of Sichuan, Sichuan University, Chengdu 610065, China; College of Biomedical Engineering/National Engineering Research Centre for Biomaterials, Sichuan University, Chengdu 610065, China; College of Biomedical Engineering/National Engineering Research Centre for Biomaterials, Sichuan University, Chengdu 610065, China; Provincial Engineering Research Center for Biomaterials Genome of Sichuan, Sichuan University, Chengdu 610065, China; College of Biomedical Engineering/National Engineering Research Centre for Biomaterials, Sichuan University, Chengdu 610065, China; Provincial Engineering Research Center for Biomaterials Genome of Sichuan, Sichuan University, Chengdu 610065, China

**Keywords:** Large Language Model, bioactive glass, relation extraction, Graph Convolutional Networks

## Abstract

Automated literature mining is key to building structured biomedical materials databases, yet current methods struggle with large publication volumes, complex entity relations and domain-specific terminology. We propose a hierarchical natural language processing (NLP) framework for extracting structured data from biomedical materials texts. Our pipeline uses named entity recognition (NER) to identify entities such as compositions, synthesis methods and properties. Sentence-level relation extraction captures direct associations (e.g. temperature, morphology), while a paragraph-level graph convolutional network (GCN) module resolves cross-sentence co-references. Rule-based templates enhance precision in specific cases. Extracted relations are integrated into a biomedical materials knowledge graph, enabling scalable and extensible data representation. Experiments show that the sentence-level model achieves 84.7% accuracy and the GCN-based module achieves 84.0%. This approach offers an efficient pipeline for structuring complex scientific texts, reducing manual effort and supporting large-scale knowledge extraction in biomedical materials and related domains.

## Graphical abstract

**Figure:**
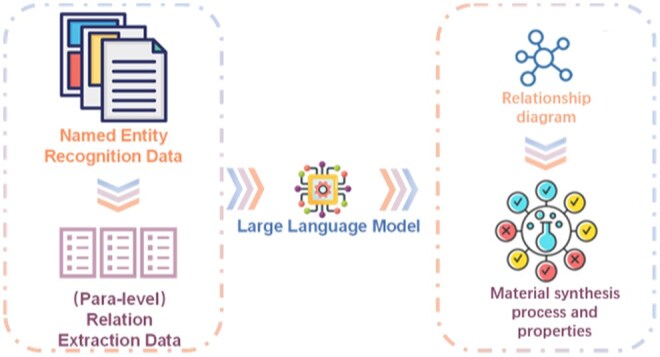

## Introduction

The acquisition of experimental data and corresponding results is fundamental to the advancement of materials science [1]. While published academic literature offers a rich source of high-quality data, most of this information remains embedded in unstructured free text [2]. Traditionally, extracting such data has relied heavily on manual efforts, which were not only time-consuming but also demand significant human, material and financial resources [3, 4]. These limitations posed a major bottleneck in large-scale data collection efforts for materials informatics [5].

To address this issue, there was a growing interest in developing automated information extraction methods capable of efficiently mining key relationships from scientific texts. Specifically, extracting the complex interactions among synthesis conditions, material compositions and properties was essential for driving progress in materials genomics [6]. However, achieving this level of automation remains challenging due to the nuanced and domain-specific language used in academic literature.

In this study, we aimed to develop a robust and efficient data extraction framework to automatically identify and construct relational knowledge particularly between synthesis conditions and material properties from large volumes of scientific literature. Our focus lay in the biomedical materials domain, where such structured knowledge is crucial for accelerating material discovery and optimization [1, 7].

In material field, artificial intelligence/machine learning (AI/ML) was widely used to assess the potential toxicity of nanomaterials, enhancing data reproducibility, improving the robustness of quantitative comparisons and promoting computational modeling and meta-analysis, thereby driving the safe-by-design approach in nanomedicine [8–11]. Furthermore, the application of AI in nanoinformatics had also attracted attention, especially in the clinical translation of nanotherapy [12]. AI also plays a crucial role in biomedical polymers, drug delivery systems, wearable electronics and smart materials, enabling the optimization of material properties, improving predictive accuracy and accelerating the research and development process [7]. For instance, AI could be used to predict the properties of polymer materials, optimize drug release kinetics, improve drug delivery system designs and develop smart responsive materials for biomedical devices [13]. It demonstrated significant value in the design, synthesis and characterization of nanomaterials [14]. Researchers used large-scale databases to develop novel nanomaterials and optimize the synthesis process through AI algorithms [5]. Additionally, AI was used to analyse characterization data of nanomaterials, improving the efficiency of information extraction and further advancing the development of biomedical nanomaterials [15].

To achieve those material predictions, a huge amount of data was needed. By using LLMs (Large Language Models) and vertical large models, the process could be significantly enhanced. Vertical large models are designed to specialize in specific domains, allowing them to handle domain-specific data more efficiently and accurately. These models could be trained on vast datasets from the materials science and nanotechnology fields, enabling them to make more precise predictions regarding material properties, performance and behavior. By combining LLMs with these domain-specific models, researchers could leverage the best of both worlds—large-scale data processing and in-depth, specialized knowledge—for more accurate and reliable material predictions. Relation extraction (RE) played a critical role in identifying the relationships between synthesis conditions, composition, structure and material properties in materials genomics research [16]. By identifying and extracting associations between specific entities, RE enabled the construction of an information network that supports materials design and performance prediction.

Graph Neural Networks (GNNs) have recently emerged as powerful tools in materials science, particularly for property prediction, reactivity modeling, inverse design and dynamic simulations [17]. By modeling molecules or crystals as graphs, GNNs capture both local atomic interactions and global structural features, improving the performance of tasks such as drug discovery, absorption, distribution, metabolism, excretion and toxicity (ADMET) property prediction and automated synthesis planning. They also offer efficient alternatives to traditional methods in molecular and excited-state dynamics.

Beyond simulation, graph-based RE methods were increasingly used to address complex entity relationships in scientific literature. Unlike sentence- or paragraph-level methods, these approaches integrate multi-layered textual information into unified graph structures, enabling cross-sentence and cross-paragraph relation modeling. This was particularly valuable in fields such as materials science, medicine and law, where texts are often long and unstructured.

Biomedical materials literature presents additional challenges due to its unstructured format and domain-specific terminology [5]. General-purpose NLP methods often lacked the adaptability required to extract reliable information from such data, limiting their effectiveness in materials databases. To overcome these limitations, we proposed a BERT-based deep learning framework that incorporates graph structures and multi-level RE.

The key contributions of our model were as follows:

- BERT-GNN integration: we combined BERT’s contextual understanding with GNN’s structural modeling to extract long-range, cross-sentence relationships between material entities.
- Paragraph-level RE (Para-level RE): specifically designed for material entities, Para-level RE captured document-level relations involving synthesis conditions, properties and other inter-entity dependencies in materials literature.
- End-to-End structuring: our pipeline automated the extraction of entities and relations—such as composition, properties and synthesis conditions—into structured data, reducing manual effort and improving efficiency.
- Cross-domain adaptability: without relying on domain-specific knowledge bases, the model generalized across diverse biomedical materials texts by focusing on functional relationships rather than specific material types.

In summary, our model addresses the limitations of existing approaches by combining graph reasoning with deep contextual embeddings, significantly improving the accuracy and scalability of data extraction in biomedical materials research.

## Relative works

Court proposed an automated data processing system based on RE, focusing on extracting key attributes related to magnetic materials from existing databases [18]. By utilizing an improved Snowball algorithm, the system could efficiently extract quadruple relations (such as Curie temperature, Néel temperature, etc) from database records and organize them into structured data. This method significantly improved the accuracy and efficiency of data extraction, providing a solid foundation for data integration and further research in the field of materials science.

Durmaz constructed an ontology in the field of materials science (such as the materials mechanics ontology) and defined various relationships between entities (such as causal, influence and associative relationships), providing a structured semantic framework for RE tasks [19]. By combining NER and RE, the system not only automated the extraction of entities from literature but also identified and extracted the complex relationships between them, aiding in knowledge discovery within materials science literature. The core of this method lay in utilizing the entity and relationship definitions within the ontology, supporting deep learning and neuro-symbolic reasoning, while also providing a foundation for building knowledge graphs in the materials science domain.

Geng proposed a rule-based iterative entity and RE method (MatIERE), which improved the accuracy and performance of para-level RE by identifying iterative entities and using material pronouns as relation triggers in materials science literature [20]. However, rule-based approaches required extensive expert annotations, which could be resource-intensive. However, traditional RE models rely on the form of relation triplets of mentioned-level named entities, which were often not suitable for processing complex biomedical texts. This was especially evident when multiple materials are stacked in the text and lack clear structure and separation, which led to confusion and failure in the RE process.

Traditional RE methods faced significant challenges, particularly in representing the ‘synthesis conditions–performance’ relationship. Existing models struggled to process complex, multi-layered information, such as how synthesis conditions—like temperature, time and pH—affect material properties, including pore size, surface area and chemical activity. Additionally, accurately defining entity boundaries and handling multidimensional information remained key difficulties for current models. Zhang *et al.* [21] proposed a graph-based method that integrates existing knowledge bases and entity relationships to enhance para-level RE, especially for complex biomedical entity relationships. However, their approach extracted relationships primarily from existing databases rather than directly from literature. BERT-GT combines BERT with graph transformers for cross-sentence RE, improving accuracy in recognizing chemical–protein relationships; however, it faces efficiency challenges when handling long texts with diverse relationship types [22]. In current document-level models, such as Gain, Autore and others [23–25], co-reference relationships needed to be explicitly specified.

However, our approach emphasized the prediction of co-reference relationships, with a particular focus on document-level RE for material entities. This distinguished our method from existing approaches in other domains, where publicly available datasets often provide explicit co-reference annotations, facilitating direct co-reference modeling as part of their prediction tasks. Such methods typically rely on pre-annotated co-reference links, thereby simplifying the extraction pipeline. In contrast, co-reference annotations were generally absent in the materials science domain, posing significant challenges for RE.

To overcome the limitations of existing methods, we integrated BERT with graph neural networks to effectively model complex cross-sentence and cross-paragraph relationships and co-reference links in biomedical materials literature, enabling end-to-end automated extraction of intricate ‘synthesis condition–performance’ relations.

Previous approaches based on rule-based systems, ontologies or shallow models heavily rely on existing databases and manual annotations, limiting their ability to handle the multi-layered complexity and co-reference of material entities. In contrast, our Para-level RE framework specifically targeted the unique material co-reference relationships in the materials domain, thereby improving extraction accuracy and robustness.

## Material and methods

We proposed a general supervised learning-based fully automated process and model for extracting entities and relationships from biomedical materials literature. Our data were sourced from 486 previously collected mesoporous bioactive glass biomedical material literature paragraphs that had completed NER. Mesoporous bioactive glass (MBG) was selected as the target material system for this study due to its structural simplicity [26], well-defined synthesis parameters, and wide application in bone tissue engineering and drug delivery. Compared to other complex biomedical materials, MBG exhibits more standardized reporting of synthesis conditions [26–28], such as precursor type, calcination temperature and pore characteristics, which improves the consistency of named entities and relations. Furthermore, its frequent appearance in high-quality literature provided rich and relatively clean training data for supervised learning models [29]. This made MBG an ideal starting point for building and validating structured information extraction pipelines.

**Figure 1.**
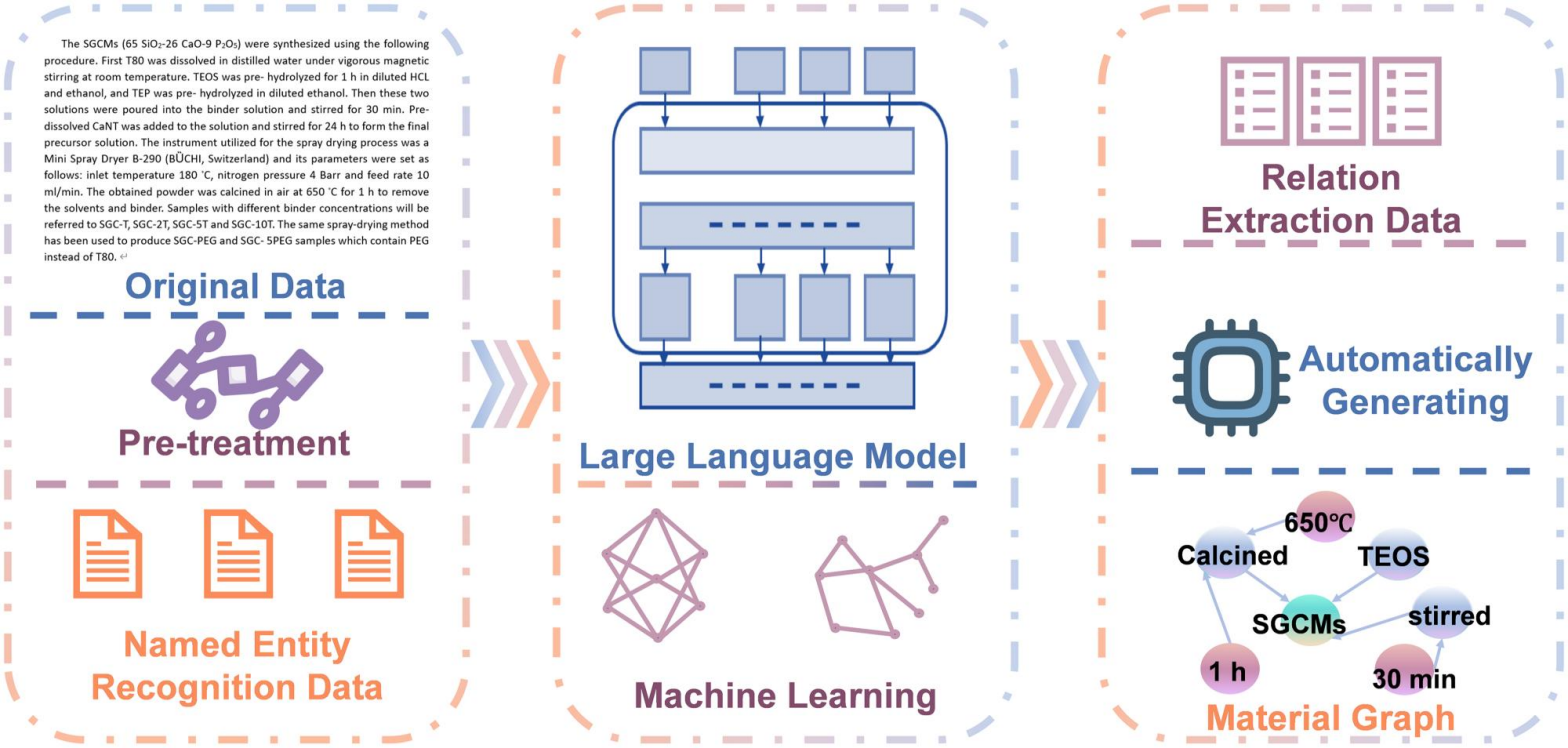
A comprehensive overview of data structuring processes.

These paragraphs were processed through a fully automated script, generating a total of 28 576 training samples, 3315 validation samples and 4732 entity-level graph datasets. Additionally, we deliberately avoided applying any data augmentation techniques to ensure the authenticity of the dataset [30]. Our approach to structuring biomedical material data involves key stages such as RE [31], para-level RE, necessary text preprocessing, rule templates [32] and script generation. Figure 1 provides an overview of the data structuring process [33]. Our model configuration effectively addresses issues such as articles containing different materials, the same condition encompassing multiple properties and varied materials sharing the same properties. By decomposing tasks, we have streamlined both the model and dataset, making them lightweight and logically clear.

### Annotation strategy

To ensure annotation quality, the 486 paragraphs were independently annotated by multiple domain experts with specialized knowledge in biomedical materials science. Annotation consistency was verified through manual review, and any discrepancies were resolved by majority voting.

### NER dataset

**Figure 2.**
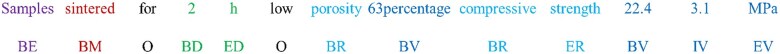
Example of NER annotation.

To ensure consistent and accurate NER annotations, we established a comprehensive set of annotation guidelines. These guidelines define the entity categories, annotation scope and criteria for boundary identification to standardize the labeling process across annotators. The detailed NER classification scheme is summarized in Table 1 and Figure 2, which outlines each entity type along with its description and annotation examples.

**Table 1.** List of NER labeling strategy

Category	Definition	Location	Phrase	Annotation example
B	Start of entities	Start of label	Bioactive glass	BE EE
I	Middle of entities	Start of label	Rate 10 C divide min	BD ID ID ED
E	End of entities	Start of label	Citric acid	BC EC
E	Material entities	End of label	BG	BE
O	Menningless words	/	Comma	O
C	Experiment condition and reagents	End of label	TEP	BC
D	All values in the experimental phase	End of label	50 g citric acid	BD ED BC BD
M	Experiment steps	End of label	Stirred at 100°C	BM O BD ED
R	material properties‘ categories	End of label	SSA	BR
V	All values of material properties	End of label	Zeta potential values 24 3 mV	BR ER O BV EV

### Relation extraction

We used an RE model to link multiple entities [34], employing the R-BERT model due to its straightforward data format [35] and achieving satisfactory results. To address data imbalance, we combined datasets created automatically by computer programming of different entity pairs for training [36], thereby enhancing the model’s accuracy and reliability while simplifying both the model and datasets.


(1) Word Embedding=Bert_last_hidden_out_put (Word)

(2) Entity Embedding=average (word embedding)⊙Node mask 

(3) Features=cat (Entity Embedding, word cls, Entity Embedding)

**Figure 3.**
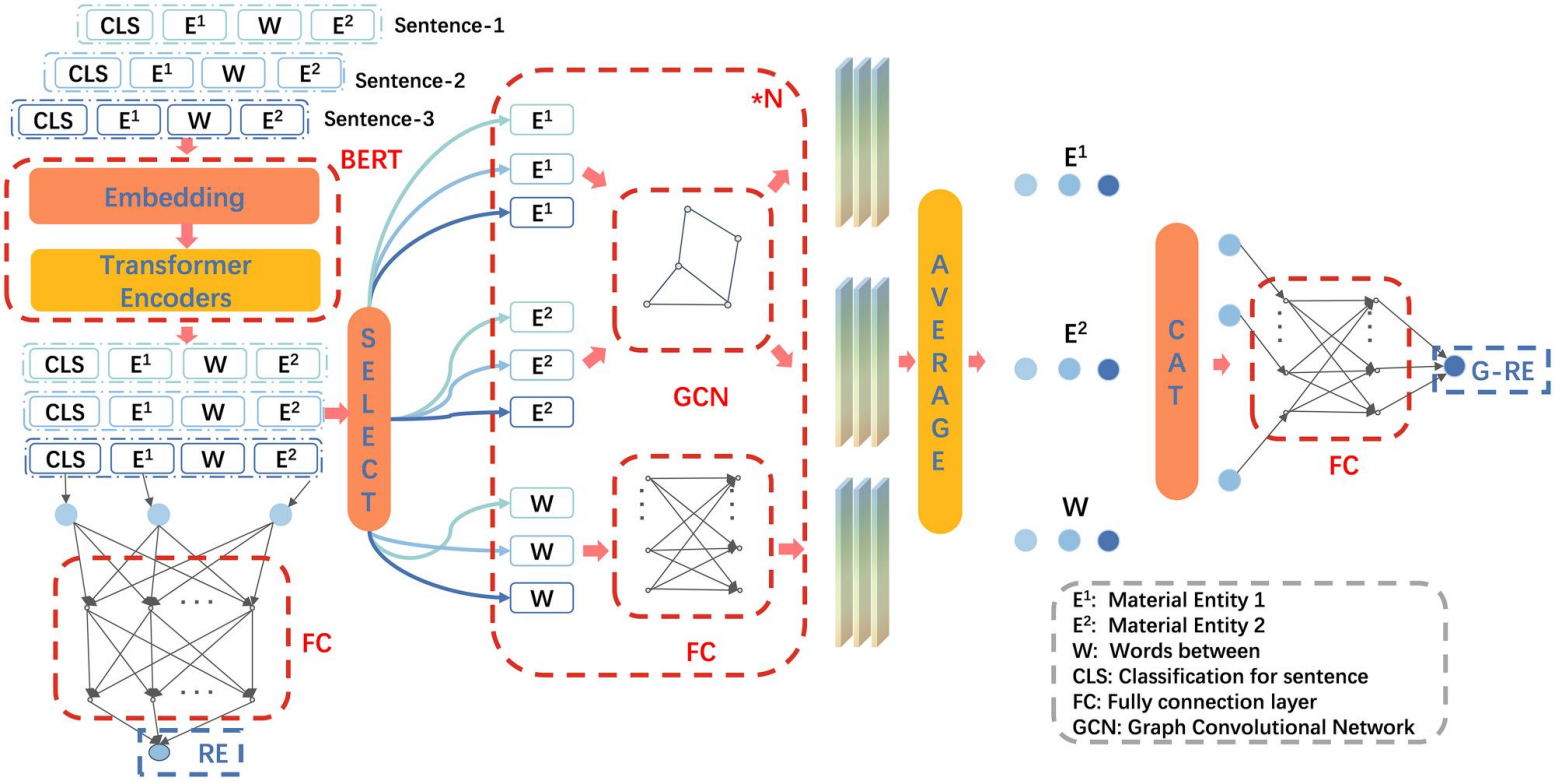
Architectural overview of the R-BERT and G-RBERT models.

Figure 3 shows the RE model structure. As shown in the figure, the R-BERT model averaged the hidden states of the CLS token and entities, then inputs them into a fully connected network for classification. This approach effectively combined global sentence-level information with local entity-level features, enhancing the model’s ability to handle relation classification tasks [37]. Compared to models that use single features, this fusion strategy better captured complex contextual relationships, thereby improving classification accuracy [38].

**Figure 4.**
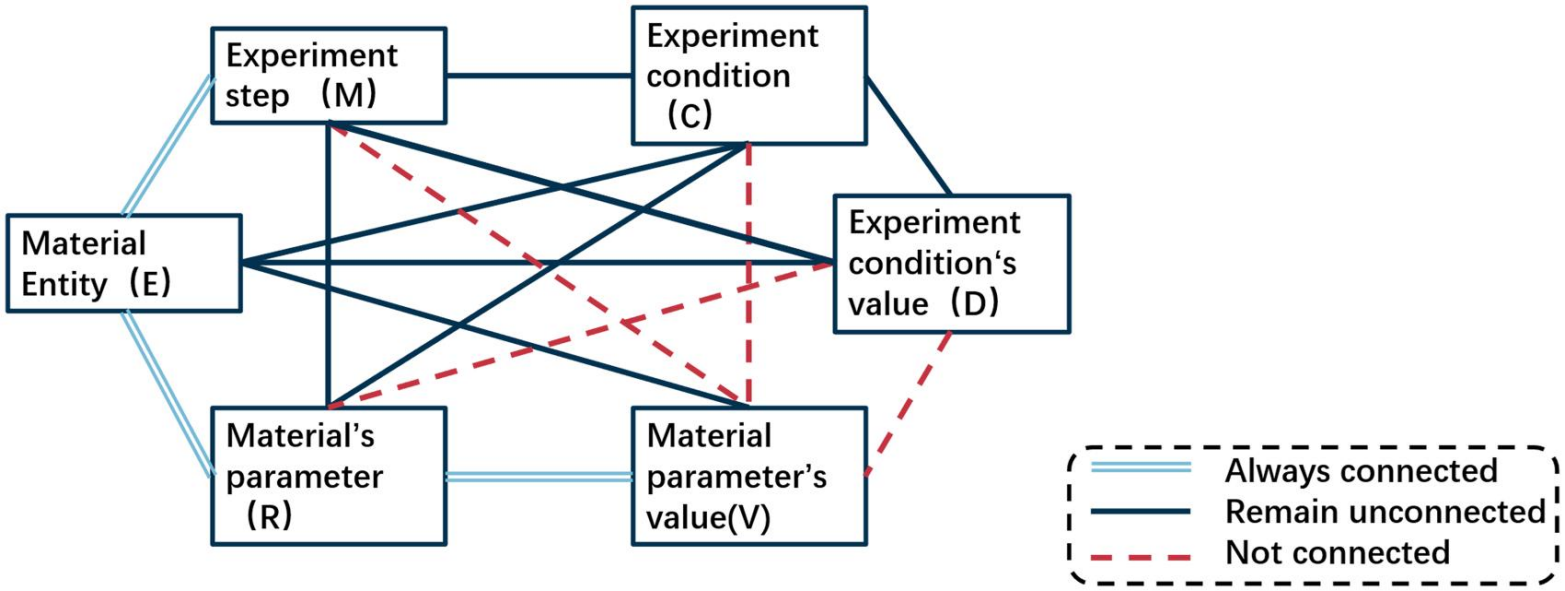
Connection’s condition between different type of entities.

In the dataset, we adopted a pairwise combination strategy, where each entity pair (e.g. EM) represents a connection between two NER categories, such as E and M. A label of 1 indicates relevance, while None indicates irrelevance. This labeling method simplifies model design and enhances scalability [39]. Based on the statistics of positive and negative samples, we observed that pairs like ER, EM and RV contained almost no negative samples(shown in Figure 4); thus, we retained all possible connections for these combinations. Additionally, based on domain knowledge, we excluded certain pairs such as MV, RD and CV, as they were not expected to be semantically connected. The binary classification approach not only reduces model complexity but also made data preprocessing and label generation more straightforward [40]. Additionally, it provided flexibility for future model expansion, allowing the architecture to remain simple when adding new categories or handling multi-label tasks.

**Figure 5.**
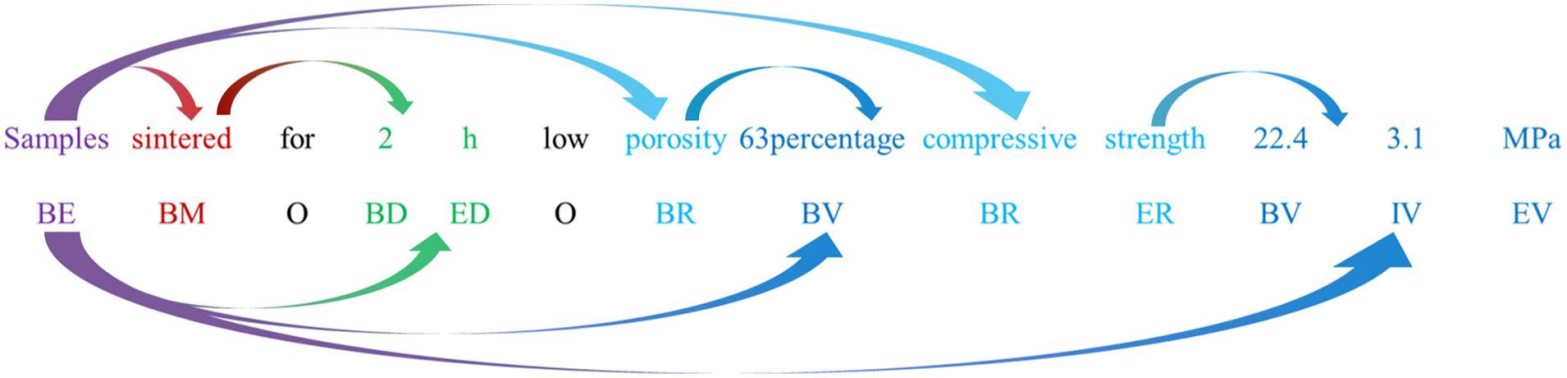
Connection’s condition between different type of entities.

**Figure 6.**
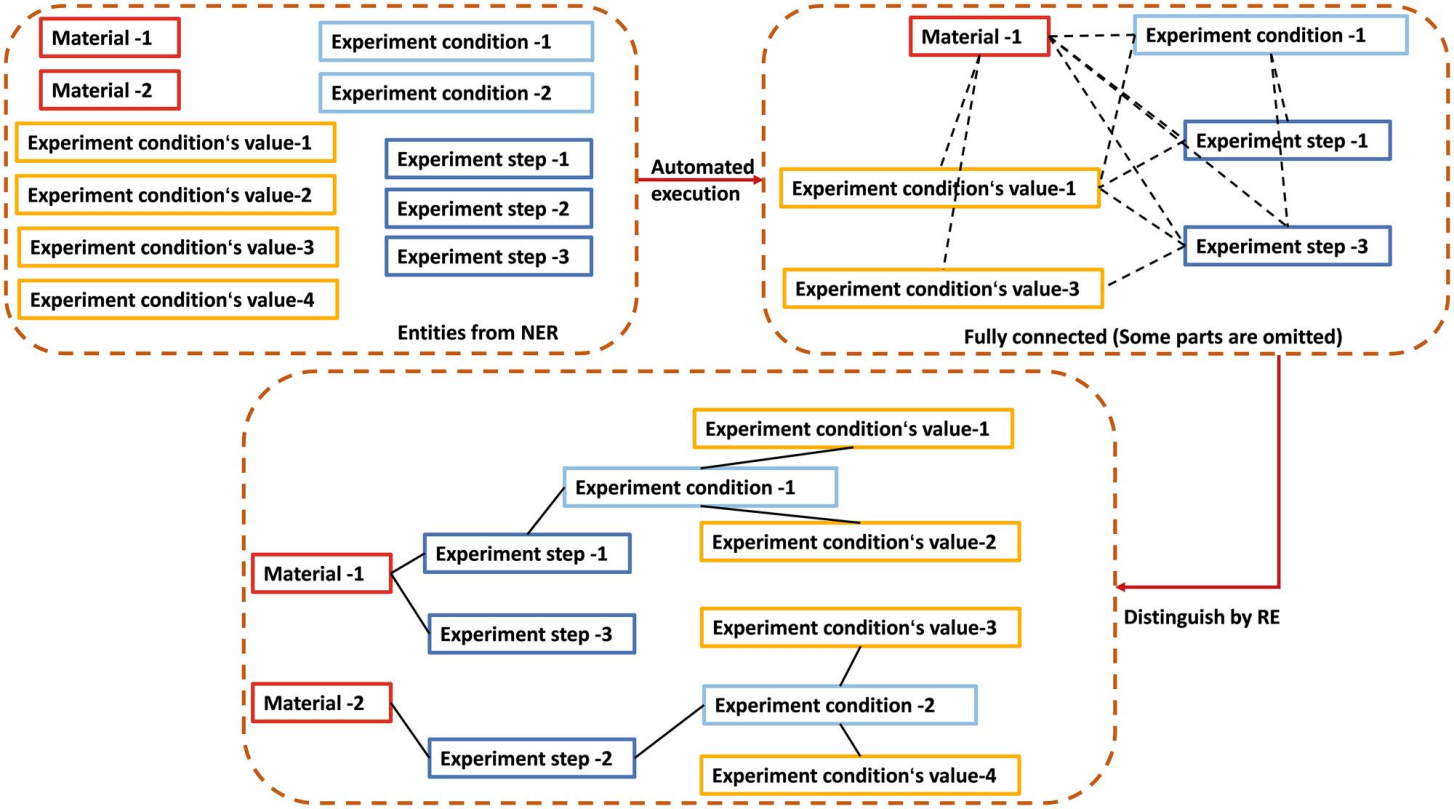
Entity graph construction based on Mention-Level relation extraction.

Given the sequential nature of experiments, we introduced an M-M (experimental step) classification. This enables the model to sequentially link each step, creating a chain of experimental steps. This design helps better understand and analyse the relationships between steps during experiments, providing additional linkage rules in the final data structuring phase. This dataset and task design enhances the model’s understanding of dynamic changes during experiments and lays the foundation for subsequent data structuring and analysis. One of the part of the schematic diagram is drawn in Figure 5, as illustrated in the workflow shown in Figure 6. NER remained a useful part of the input data for the RE task. This approach prevented the illustration from becoming overly complex while ensuring that the model retained access to complete contextual information.

### Paragraph-level relation extraction

Due to the length of the article and model limitations, para-level RE was adopted, followed by merging the data of materials with the same name to achieve comprehensive material data collection across the entire document.

In biomedical material texts, merely classifying entity mentions and other attributes is inadequate. It was common to use general terms that encompass specific materials or to refer to materials by other names (e.g. solution) before they are produced. Therefore, classifying different material names within the same paragraph was essential for creating complete structured data [41, 42]. However, for other entities, document-level RE was not necessary, as they inherently represent distinct entities within material literature, and their relationships could be identified at the sentence level. Furthermore, we predicted relationships between entities that are not the same name, which made models using datasets like DOCRE [43] unsuitable for our task. The DOCRE dataset directly provided relationships between material entities predicted at the paragraph level, whereas our focus was on predicting relationships between distinct, non-identical entities across different contexts. The exact location of the defining relationship between entities in the text is often unclear. In such cases, a single RE could not capture the relationships between entity pairs. Therefore, we introduced GCN for relationship extraction. In a GCN, each entity mention is considered a node, and edges between entities were formed based on their alternating appearances in the text. We developed a GCN-based model to effectively capture semantic relationships between entities, searching for relationships across the entire graph through each edge [44].

The exact location of the defining relationship between entities in the text is often unclear [45]. In such cases, a single RE could not capture the relationships between entity pairs. As evidenced by the subsequent results, the RE training failed when solely employed to extract relationships between material entities. Therefore, we introduced GCN for relationship extraction. In a GCN, each entity mention was considered a node, and edges between entities were formed based on their alternating appearances in the text. We developed a GCN-based model to effectively capture semantic relationships between entities, searching for relationships across the entire graph through each edge.

The dataset defined four labels to represent the relationships between two material entities: (1) Material 1 includes Material 2, (2) Material 2 includes Material 1, (3) Unrelated and (4) Identical. Accordingly, we used the results of NER, RE and para-level RE as the foundation for scripting and generating structured data and data structure diagrams for biomedical materials.

The para-level RE model comprised several steps. First, our model read material nodes and edges where two materials alternate from the para-level RE dataset as part of the model input. Additionally, paragraph information from the original NER dataset was used as another part of the model input. Based on these nodes and edges, the model constructs three graphs: the first graph included all mentions of Material 1, the second graph included all nodes of Material 2 and the third graph represented the alternating edges between Material 1 and Material 2. In these graphs, all nodes and edges were initially masked as 1 (relevant) or 0 (irrelevant) based on their start and end in the original NER dataset. After BERT embedding, the original representations of nodes and edges were obtained


(4) Word Embedding= Bert_last_hidden_out_put(Word)

(5) $$\mathrm{Node}\;\mathrm{Embedding}=[\;\begin{array}{c} W\mathrm{ord}\;\mathrm{Embedding}\\ \ldots \ldots \\ W\mathrm{ord}\;\mathrm{Embedding} \end{array}]⊙\;\mathrm{Node}\;\mathrm{mask}$$

Next, because nodes and edges have variable lengths, we used averaging to obtain 768-dimensional fixed-length feature vectors. We summed and averaged the Node Embedding according to the Node mask length to get a 768-dimensional node representation. The same process was applied to Edge Embedding.

### GCN and edge average


(6) $$H_{\mathrm{node}}^{n}=\sigma \left({{\hat{D}}^{-\frac{1}{2}}\hat{A}}^{\;}{\hat{D}}^{-\frac{1}{2}}H_{\mathrm{node}}^{n-1}W\right)$$

(7) $${\;H}_{\mathrm{edge}}^{n}=\sigma ({W*H}_{\mathrm{edge}}^{n-1}+b)$$

The model updated nodes of Entity 1 and Entity 2 using a GCN with shared weights. After each layer update, the node’s overall average is taken as part of the final classification features. These nodes were then reentered into the GCN for further updates, repeating the averaging process. This design allowed the model to iteratively integrate and update node features through multiple GCN layers, summarizing these features by averaging them to form the final classification features.

**Figure 7.**
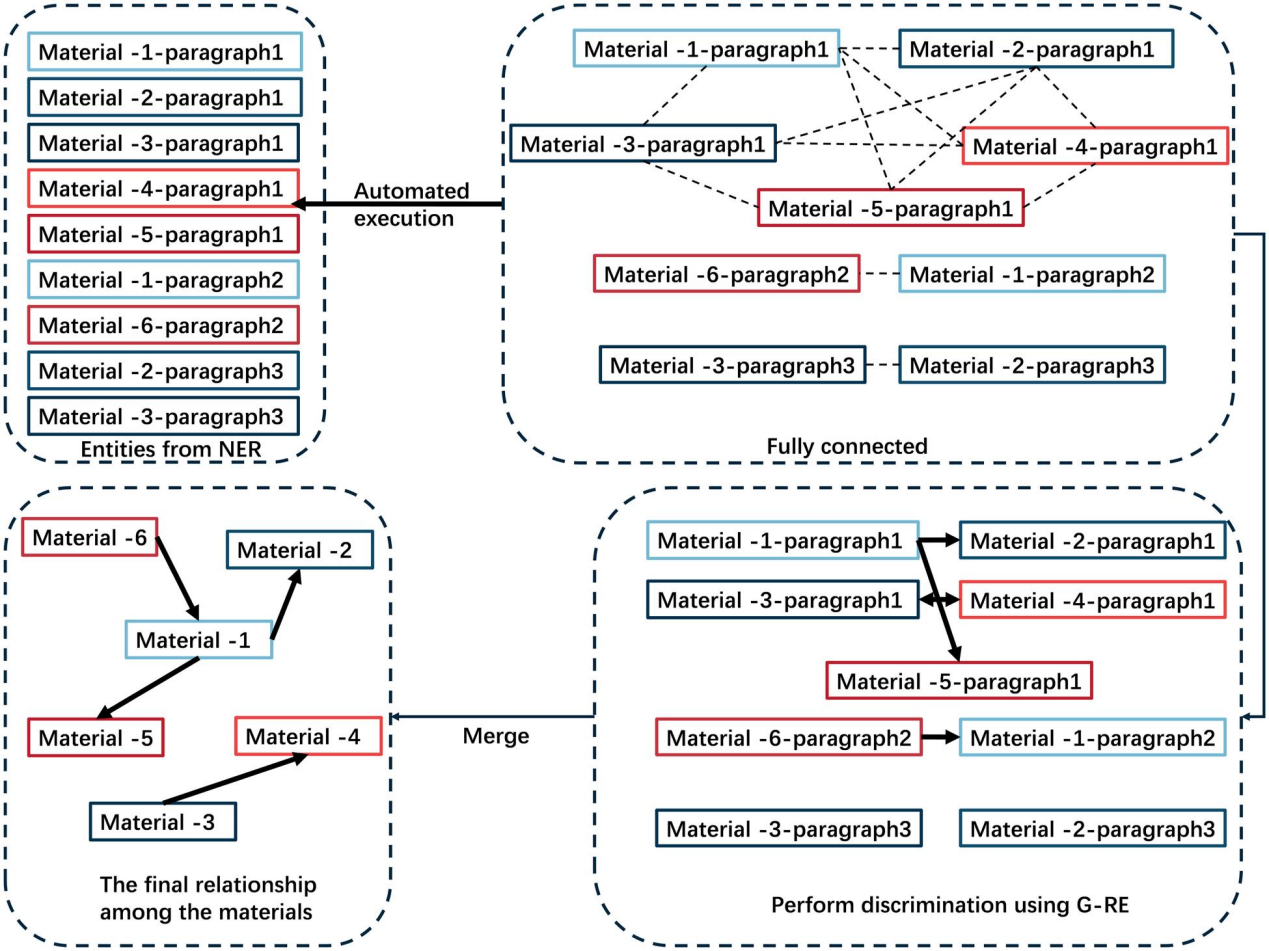
Inter-entity relationships among material entities at the paragraph level.

During the classification phase, we utilized the original features along with features from each stage of node convolution layers and edge linear transformations [46]. This approach provided richer and more abstract representations of nodes and edges, enabling better capture of relationships between nodes and edge connections, thereby enhancing the performance of the para-level RE task [47]. As shown in Figure 7, materials were connected by our model. Single arrows represent the material relationships of general or synonymous name, while double arrows represent the same material relationship


(8) $$H=\mathrm{cat}\left(H_{\mathrm{node}1}^{\;\mathrm{ave}},\cdot \cdot \cdot \cdot \cdot \cdot ,\;H_{\mathrm{edge}}^{n-\mathrm{ave}},\;H_{\mathrm{node}2}^{\;\mathrm{ave}},\cdot \cdot \cdot ,\;H_{\mathrm{node}1}^{n-\mathrm{ave}},H_{\mathrm{edge}}^{\;\mathrm{ave}},\;H_{\mathrm{node}2}^{n-\mathrm{ave}},\right)$$

(9) Result=softmax(W*H)

### Data structuring strategy

**Figure 8.**
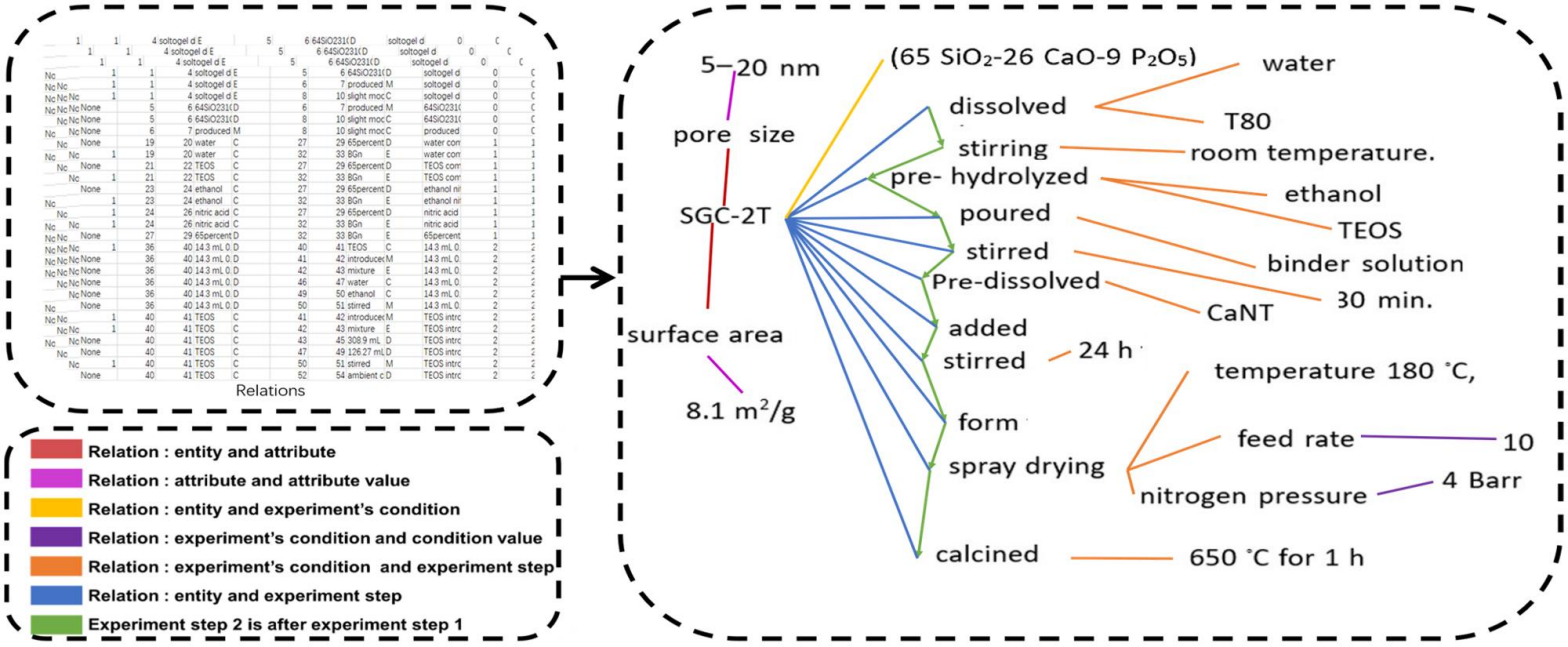
Representative example of experimental data visualization for material characterization.

As shown in Figure 8, material entities were initially structured into graphs. In order to achieve this, we developed a series of scripts for structuring mesoporous bioactive glass materials. First, we: (1) connected all MC and MM relationships to form an initial linked list structure; (2) linked MD relationships and C-type entities in the list; (3) connected remaining C-type entities to E-type entities based on EC relationships; (4) connected the final M position in EM relationships to the MM list, retaining C branches in the MM list where EC, MC and EM relationships coexist; (5) Formed ERV branches when ER, EV and RV relationships existed; otherwise, connected pairs directly. Next, we used para-level RE results to merge material entity graphs. Specifically, we first merged material structure graphs for pairs of entities classified as SAME, copying all branches between the two entities to form a new material graph. For entities classified as general-specific, we assigned branches of the general material graph to the specific material graph without copying conflicting nodes and edges. For instance, when copying ECD branches, if the specific material graph has the same EC but different CD, we discard the branch. Finally, we performed another merge of SAME-classified entity material structure graphs. This resulted in a final paragraph material structure graph, which could be easily stretched into a one-dimensional list based on topological relationships and stored in a database or table. We linked material graphs appearing in different paragraphs in sequence using a simple rule of material name matching.

In summary, we deconstructed each paragraph containing data from a paragraph into entities as nodes, RE results as linking edges and experimental step chains. The paragraph-level RE results were used to merge multiple incomplete abstract subgraphs, stored separately in different datasets. These datasets preserved various abstract structural information.

## Results

### Training–testing split of the dataset

The original NER dataset was randomly split using a 10:1 ratio, and corresponding RE and para-level RE datasets were created based on this split, ensuring that the validation set did not overlap with the training set.

### Base model selection and optimizer

In this study, the BERT-BASE-UNCASED model was used for word embedding, with AdamW as the optimizer [48]. Further, two domain-specific baseline models were additionally selected for further training, as our model was compatible with various pre-trained baseline models.

### Performance metrics

Accuracy, *F*1-score, precision and recall were used as evaluation metrics for our model [49]. The specific calculation formulas are as follows.

Matthews correlation coefficient (MCC) measures the overall quality of binary classifications, while True Negative Rate (TNR), also known as specificity, evaluates the proportion of correctly identified negative samples


(10) $$\mathrm{ACC}=\frac{\mathrm{TP}+\mathrm{FP}}{\mathrm{TP}+\mathrm{FP}+\mathrm{TN}+\mathrm{FN}}*100\%$$

(11) $$P=\frac{\mathrm{TP}}{\mathrm{TP}+\mathrm{FP}}$$

(12) $$R=\frac{\mathrm{TP}}{\mathrm{TP}+\mathrm{FN}}$$

(13) $$F1=\frac{2PR}{P+R}$$

The accuracy of the model’s results was entirely judged by human experts.

### NER model performance

**Figure 9.**
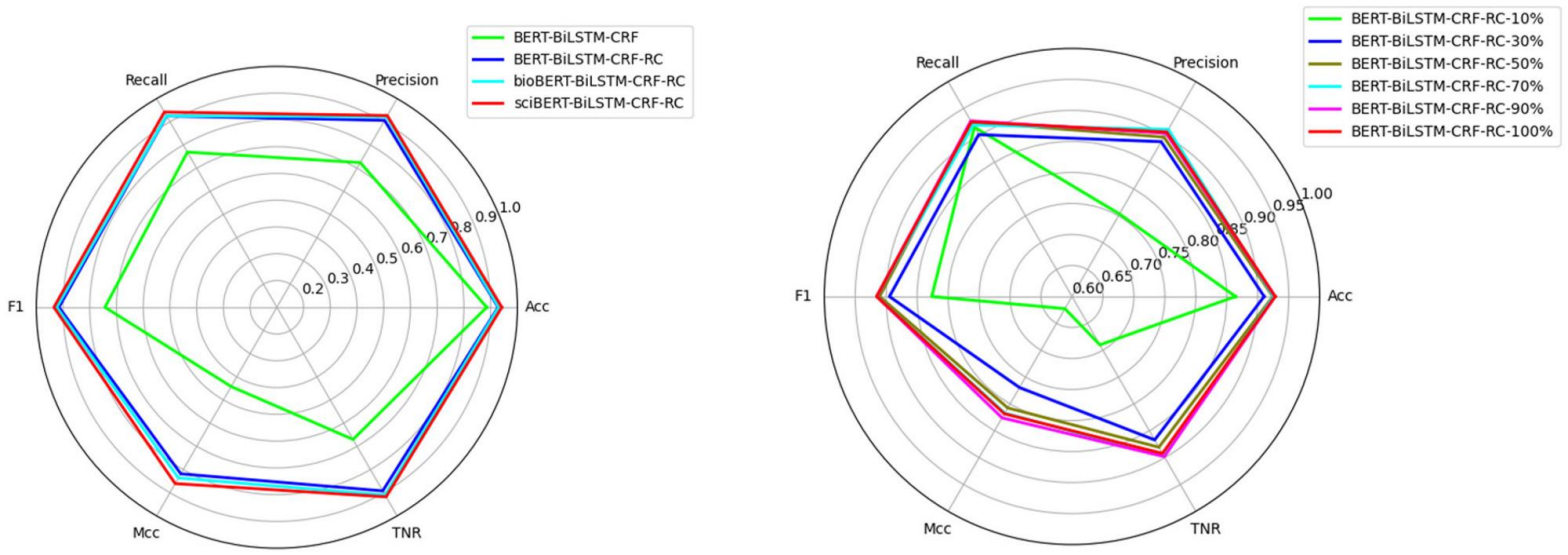
Performance evaluation of the NER model on biomedical materials literature.

The results of the NER model are presented in Figure 9. In our previous work, we applied a reading comprehension (RC) approach to enhance the NER process, which was evaluated on datasets ranging from 10% to 100% of the full data. This RC-based method consistently outperformed the baseline across all evaluation metrics, demonstrating its effectiveness in improving NER performance. Building on this prior success, the present study further investigates para-level RE to explore deeper entity-level associations.

### RE model performance

We directly employed the R-BERT model for RE and achieved an *F*1-score of 84.6%.

**Figure 10.**
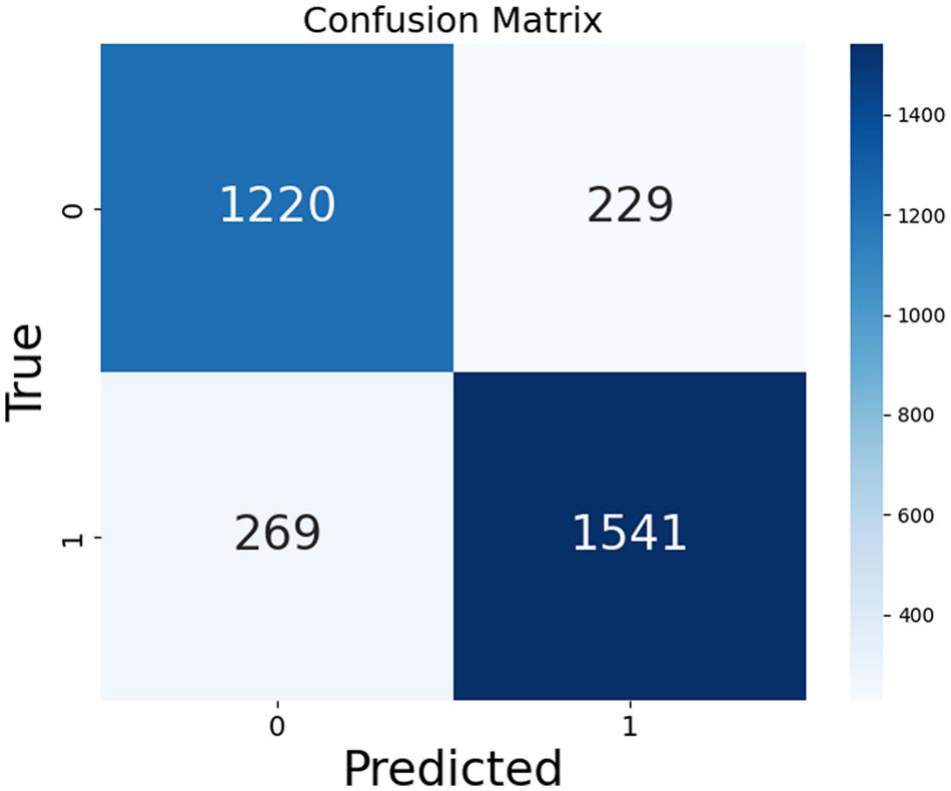
Performance evaluation via confusion matrix for relation extraction.

More detailed NER and RE metrics are provided in Table 2 and Figure 10.

**Table 2.** NER and RE models’ performance

	Actuary	Precision	Recall	*F*1
NER	0.942	0.927	0.942	0.934
RE	0.847	0.845	0.847	0.846

### Para-level RE model performance

Table 3 summarizes the performance of different para-level RE models. Among all compared methods, our bioBERT-based model achieved the best overall performance, with a precision of 0.840, recall of 0.834 and an *F*1-score of 0.818, followed closely by our BERT and sciBERT variants. In contrast, the Gain model showed considerably lower performance, with an *F*1-score of 0.696. Notably, the standard RE model failed to converge during training, resulting in no valid precision, recall or *F*1-score, which further highlights the challenges of directly applying traditional RE methods to this task.

**Table 3.** Para-level RE models’ performance

	Actuary	Precision	Recall	*F*1
Gain	0.650	0.649	0.750	0.696
R-BERT	Nan	Nan	Nan	Nan
Ours-BERT	0.825	0.836	0.780	0.799
Ours-sciBERT	0.824	0.851	0.772	0.795
Ours-bioBERT	0.840	0.834	0.808	0.818

### Analysis of RE results

The higher error rates mainly occur in the EV, CD and ED categories. EV involves performance indicators and experimental results with comparative or speculative expressions (e.g. ‘improved’, ‘slightly lower’) (Table 4), and its relatively small sample size further limits the model’s generalization, making relation judgment difficult. CD contains complex chemical entities, leading to confusion between similar components (e.g. CaO and Ca(OH)_2_). ED covers diverse experimental conditions (e.g. ‘at 37°C’, ‘37°C incubation for 24 h’), which the model struggles to generalize. In contrast, EC and ER are expressed with standardized terms or simple physical attributes, making them easier to recognize.

### Analysis of para-level RE results

**Figure 11.**
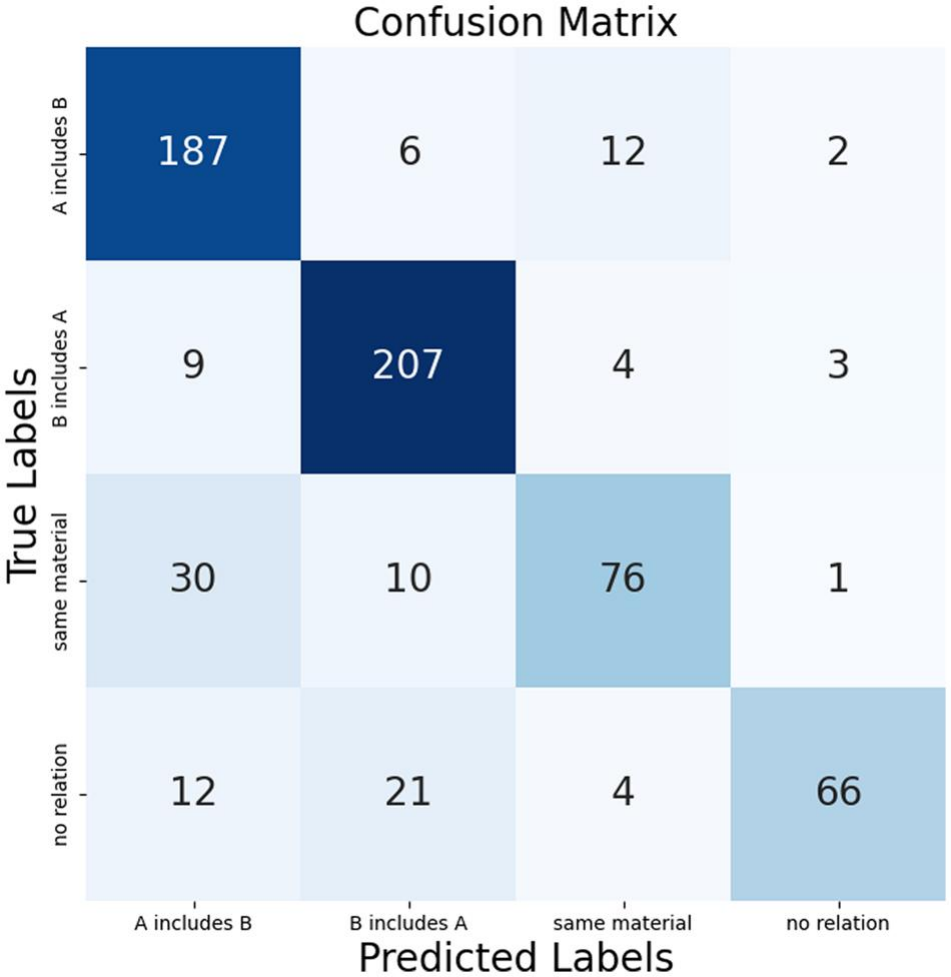
Performance evaluation via confusion matrix for paragraph-level relation extraction.

The confusion matrix (Figure 11) shows high overall accuracy, with inclusion relations predicted most accurately. Major errors occur in the ‘same material’ and ‘no relation’ categories. ‘Same material’ is frequently misclassified as inclusion (30 and 10 cases), likely due to semantic overlap with compositional descriptions, while ‘no relation’ is often mislabeled as inclusion (12 and 21 cases), reflecting the semantic ambiguity of material entity relations in literature and the diversity of negative samples.

## Discussion

MBG has emerged as a representative material in biomedical research due to its consistent terminology, controllable experimental parameters and structured formatting [26] (see Table 4). It demonstrates broad applicability in areas such as bone regeneration, tissue scaffolding and therapeutic ion release [50, 51]. Compared to other emerging materials, these characteristics significantly reduce noise in text mining processes, making MBG an ideal testbed for developing and validating robust entity and RE models [52].

**Table 4.** Error statistics across different relation types

Relation type	Total	Errors	Error rate (%)
EV	18	6	33.33
CD	121	36	29.75
ED	62	16	25.81
EC	184	29	15.76
ER	25	3	12.00

This study leverages literature from ScienceDirect and integrates a multi-module NLP approach to achieve structured extraction from biomedical materials texts. The RE model effectively identifies interactions among entities such as materials and experimental conditions, while the para-level RE captures cross-sentence relations through global context reasoning, thereby supporting semantic integrity in constructing high-quality datasets [53, 54]. Unlike conventional methods that rely on external evidence, our approach autonomously discovers latent relations including inclusion, coordination and non-identical entity associations, demonstrating strong adaptability and transferability [55].

However, the current model primarily focuses on physicochemical properties and synthesis conditions, without incorporating *in vivo* and *in vitro* experimental data. This limitation partially restricts the model’s comprehensive applicability in biomedical scenarios. Future work should aim to develop NER datasets encompassing experimental research to broaden the model’s scope and further enhance its generalization ability in complex biomedical contexts.

### Transferability

**Figure 12.**
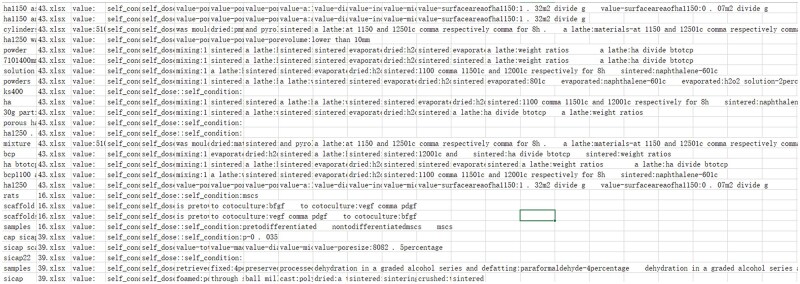
Fully automated workflow for NER, relation extraction and temporal alignment of experimental procedures.

The entire process is fully automated. As illustrated in Figure 12, once the outputs for NER, RE and para-level RE are generated, the model automatically applies a post-processing script to derive the corresponding values and organize them into the previously defined data schema. Although the complete removal of non-material entities (e.g. intermediate products and aliases) cannot be entirely guaranteed, the system autonomously arranges experimental procedures in chronological order and accurately links them to their respective material entities. This fully automated temporal alignment provides a clearer representation of material-related experimental workflows, thereby improving the interpretability and expanding the applicability of the structured data for downstream biomedical analyses.

## Conclusion

In conclusion, by flexibly adjusting predefined structuring scripts, our model can adapt to diverse material-related literature, providing researchers in various fields with efficient and accurate tools for literature processing and analysis. This flexibility and transferability offer broad application prospects and open new avenues for materials science research and applications. Overall, the proposed fully automated output method efficiently and accurately converts prediction results into structured tables, establishing a reliable foundation for subsequent analysis, research and applications. Manual curation is required only when necessary, during downstream data processing to eliminate redundant non-material entities, while the entire workflow remains predominantly automated. This design substantially improves efficiency, reduces human errors and enhances research reproducibility and scalability, thereby contributing to the advancement of large-scale, data-driven materials research.

## Data Availability

The data that support the findings of this study are derived from publicly available table dataset from PubTabNet and literature on ScienceDirect, obtained by searching with the keyword ‘mesoporous bioactive glass’.
